# Directional Sensitivity of a MEMS-Based Fiber-Optic Extrinsic Fabry–Perot Ultrasonic Sensor for Partial Discharge Detection

**DOI:** 10.3390/s18061975

**Published:** 2018-06-20

**Authors:** Wenrong Si, Chenzhao Fu, Delin Li, Haoyong Li, Peng Yuan, Yiting Yu

**Affiliations:** 1 State Grid Shanghai Electric Power Research Institute, Shanghai 200437, China; siwenrong@126.com (W.S.); 13512111246@139.com (C.F.); 2 Key Laboratory of Micro/Nano Systems for Aerospace (Ministry of Education), Northwestern Polytechnical University, Xi’an 710072, China; delinli@mail.nwpu.edu.cn (D.L.); lyhaoyong@mail.nwpu.edu.cn (H.L.); 3 Shaanxi Province Key Laboratory of Micro and Nano Electro-Mechanical Systems, Northwestern Polytechnical University, Xi’an 701172, China; 4 Xi’an Maorong Power Equipment Co., Ltd., Xi’an 710048, China; y5anpeng@126.com

**Keywords:** fiber-optic sensors, Fabry–Perot, ultrasonic sensor, partial discharges

## Abstract

Extrinsic Fabry–Perot (FP) interferometric sensors are being intensively applied for partial discharge (PD) detection and localization. Previous research work has mainly focused on novel structures and materials to improve the sensitivity and linear response of these sensors. However, the directional response behavior of an FP ultrasonic sensor is also of particular importance in localizing the PD source, which is rarely considered. Here, the directional sensitivity of a microelectromechanical system (MEMS)-based FP ultrasonic sensor with a 5-μm-thick micromechanical vibrating diaphragm is experimentally investigated. Ultrasonic signals from a discharge source with varying incident angles and linear distances are measured and analyzed. The results show that the sensor has a 5.90 dB amplitude fluctuation over a ±60° incident range and an exciting capability to detect weak PD signals from 3 m away due to its high signal–noise ratio. The findings are expected to optimize the configuration of a sensor array and accurately localize the PD source.

## 1. Introduction

Fiber-optic extrinsic Fabry–Perot interferometric (EFPI) sensors based on sensitive vibrating diaphragms have been studied extensively due to their distinct advantages of high sensitivity, compact size, versatility, and immunity to electromagnetic interference [1]. These sensors have been successfully applied for sensing acoustic waves, vibrations, temperatures, pressures, refractive indexes, and strain [2]. The diaphragm, as one of the reflecting mirrors constructing the Fabry–Perot (FP) cavity, is critical to the sensor’s performance and especially its sensitivity. Different materials have been employed to fabricate a highly responsive diaphragm, including silicon [3,4], silver [5,6], polymer [7,8,9,10], and graphene [11,12]. Our previous work [13] showed that a thinner diaphragm with a smaller radius will result in a larger deformation at a determined resonant frequency; thus, such diaphragms show promise for detecting a weak signal. Moreover, the rapidly developing microelectromechanical system (MEMS) technology provides extra advantages in diaphragm fabrication, component alignment and integration, and high-volume fabrication potential based on the mature semiconductor industry [3,4,14,15]. By utilizing the MEMS technology and a silicon-on-insulator (SOI) wafer, the thickness of the sensing diaphragm can thus be precisely controlled [13].

Precisely monitoring the ultrasonic wave generated by the partial discharge (PD) in the electrical equipment, and furthermore localizing it, is of great importance for preventing the disastrous failure of a power system [16,17,18]. Thus, the ultrasonic sensors should possess a linear response to the pressure variation and be sensitive to a low acoustic pressure within a wide incident range [19]. On the other hand, the bandwidth of ultrasonic sensors was suggested to cover the frequency range of 40–300 kHz [19,20] by considering the propagating characteristics of sounds and also the environmental noises. However, there is a trade-off between the direction-dependent sensitivity and an ultrasonic resonant frequency, both of which are determined by the geometrical dimension of the sensing diaphragm. An ultra-sensitive silver diaphragm of 67.01 nm/Pa may limit the ultrasound response because of a small resonant frequency at 3 kHz [4], while an ultrasonic sensor that has a 3.9 nm/kPa sensitivity and a 242 kHz resonant frequency may fail to detect the low acoustic pressure and response over a large incident range [21]. Thus, it is important to fabricate the diaphragm to have high sensitivity in the PD-induced ultrasonic frequency range. Furthermore, a sensing diaphragm is expected to have a relatively flat response within the wide incident angles of an ultrasonic wave [19]. This flat angular response is beneficial for accurate localization and a reduced sensor array, but has been, however, rarely considered in previous publications. In addition, the direction-dependent response of the sensors will be also influenced by the FP cavity alignment manner and packaging materials. Therefore, it is necessary to make an experimental investigation.

In this paper, the directional sensitivity based on the different incident angles and linear distances of the proposed highly sensitive MEMS-based EFPI ultrasonic sensor are measured and analyzed in the time domain. With the intensity interrogation, the output voltage is varied according to different PD locations. Then, a Fast Fourier Transform (FFT) is performed to investigate the frequency response and determine the signal–noise ratio (SNR) of the whole sensing system. The results show that the sensor is sensitive enough to detect a weak signal from all angles within 1 m and offers the possibility for locating the PD within a simplified sensor array. Moreover, this experimental investigation can be used to design the sensor package as well as the sensor array configuration.

## 2. Working Principle

**Figure 1:**
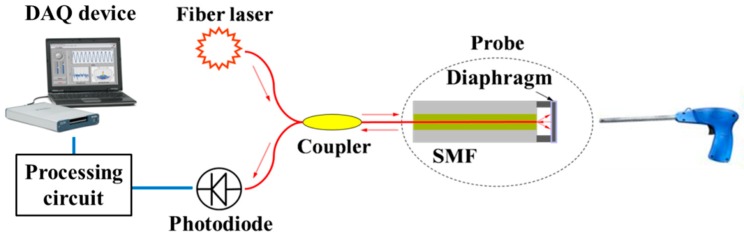
Schematic of the proposed ultrasonic sensing system. DAQ, data acquisition; SMF, single mode fiber.

The air-coupled ultrasonic fiber sensing system for non-contact PD detection is shown in Figure 1. The light source centered around 1550 nm with a narrow bandwidth of 0.1 nm from a distributed feedback (DFB) fiber laser is sent to the ultrasonic sensor probe through an optical coupler. The incident light transmits along the single mode fiber (SMF) and comes into the FP cavity between the fiber’s end face and the diaphragm covered with a gold film on an SOI chip. Then, the interference between the reflected lights at two cavity mirrors is generated, whose intensity is modulated by the vibration of the diaphragm *y*(*p*) corresponding to the varying acoustic pressure *p* [13],

(1) $$y(p)=\frac{3(1-\mu^{2})p}{16Eh^{3}}R^{4}$$

where *h* and *R* are the diaphragm thickness and radius, respectively, and *E*, *μ*, and *ρ* are the elastic modulus, Poisson’s ratio, and density of the diaphragm material, respectively. A photodiode collects the interfered light and converts it into the electrical current. After the signal is amplified and filtered by a processing circuit, the voltage signal *V*_out_ is then acquired by a data acquisition (DAQ) device, which is given by [22]:

(2) $$V_{\mathrm{out}}\propto P_{l}\cdot G\cdot R_{\mathrm{FP}}$$

where *P_l_* is the power emitted from the laser source and *G* is the gain of the photodetector. The reflectance of the FP cavity *R*_FP_ is the ratio of the reflected power to the incident power [23], which can be expressed as:

(3) $$R_{\mathrm{FP}}=\frac{R_{1}+R_{2}+2\sqrt{R_{1}R_{2}}\cos\phi }{1+R_{1}R_{2}+2\sqrt{R_{1}R_{2}}\cos\phi }$$

where *R*_1_ and *R*_2_ are the reflectivity of each mirror and *ϕ*, the round-trip phase difference, is defined as:

(4) $$\phi =\frac{4\pi nL}{\lambda }$$

where *n* denotes the refractive index of the FP cavity, *L* is the cavity length, and *λ* represents the free-space wavelength. With the intensity interrogation, the voltage amplitude indicates the interfered light intensity modulated by the varying acoustic pressure. Because the acoustic pressure is exponentially decayed during the spreading of the ultrasonic wave, *V*_out_ will be changed by the different distances and orientations from the discharge source to the sensor.

## 3. Sensor Design and Fabrication

The diaphragm design of the EFPI ultrasonic sensor was presented in our previous work [13]. For an intact diaphragm, two geometrical parameters of thickness *h* and radius *R* are involved in designing the sensing structures; both determine the fundamental frequency *f* and the central deformation *y*(*p*) of the diaphragm. Normally, the larger central deformation *y*(*p*) means a higher response sensitivity. By multiplying *f* and *y*(*p*), there follows:

(5) $$f\cdot y(p)=\alpha \frac{h}{R^{2}}\cdot \beta \cdot p\frac{R^{4}}{h^{3}}=\alpha \beta \cdot p{\left(\frac{R}{h}\right)}^{2}$$

where *α* and *β* are the equivalent coefficients influenced by the elastic modulus, Poisson’s ratio, and density of the diaphragm material. Thus, finding the values of *R* and *h* that cause the maximal central deformation *y*(*p*) at a certain frequency *f* is equivalent to searching for the maximal ratio of *R/h*. To retain a certain frequency, the thickness and the radius change, ∆*h* and ∆*R*, should satisfy the equation:

(6) $$\frac{h+\Delta h}{{\left(R+\Delta R\right)}^{2}}=\frac{h}{R^{2}},\;\mathrm{or}\Delta h=\frac{h[2R\Delta R+{\left(\Delta R\right)}^{2}]}{R^{2}}.$$

Then, Equation (7) can be obtained:

(7) $$\frac{R+\Delta R}{h+\Delta h}=\frac{R}{h}\cdot \frac{R^{2}+R\Delta R}{R^{2}+2R\Delta R+{\left(\Delta R\right)}^{2}}<\frac{R}{h}$$

therefore, it comes to the conclusion that a thinner diaphragm with a smaller radius will result in a larger deformation at a determined resonant frequency. In this work, a 5-μm-thick diaphragm is used to improve the sensitivity, which is thinner than many other EFPI ultrasonic sensors [9,21]. According to the simulation results of a constant resonant frequency of 60 kHz and the thickness of 5 μm, the diameter of a silicon diaphragm is then determined to be 1120 μm, which contributes to a high sensitivity of 733 nm/kPa.

**Figure 2:**
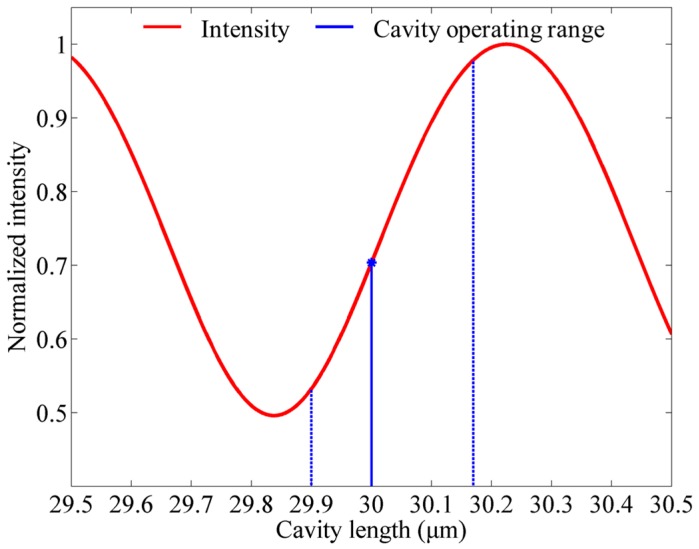
Intensity response of Fabry–Perot (FP) cavity.

Figure 2 gives a simulation result of the relationship between cavity length and the interference intensity according to Equation (3), where *R*_1_ = 0.04, *R*_2_ = 0.9, and *λ* = 1550 nm. The figure shows that the detected light intensity varies in the period of *λ*/2. To obtain the linear range of the intensity variation, the initial cavity length should be set in the middle of a *λ*/4 period to provide an adequate operating range for the FP cavity. Therefore, a 30-μm-deep cavity is designed in this work.

**Figure 3:**
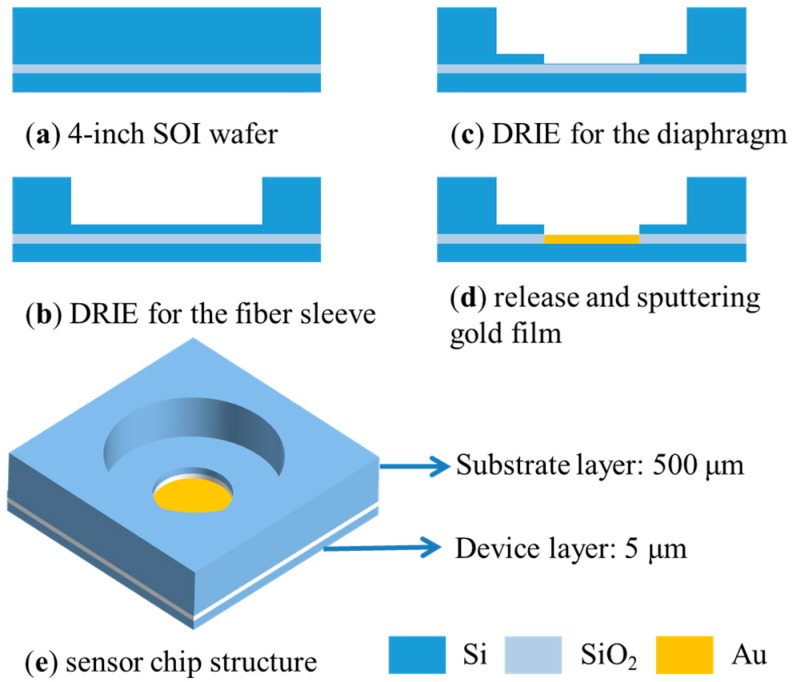
The schematic of fabrication process and the sensor chip structure. SOI, silicon-on-insulator; DRIE, deep reactive ion etching.

The detailed fabrication process of the sensor chip is illustrated in Figure 3. Firstly, the 500-μm-thick substrate layer of the SOI wafer is patterned by photolithography, and a deep hole for the fiber sleeve is fabricated by utilizing the deep reactive ion etching (DRIE) process. After the second pattern is transferred, a stepped hole with a depth of 30 μm is etched and stopped at the SiO_2_ layer to form the FP cavity and the vibrating diaphragm as shown in Figure 3c. Then, the diaphragm is released into buffered hydrofluoric acid and a gold film is sputtered on the inner face of the diaphragm to form the reflective surface of the FP cavity.

**Figure 4:**
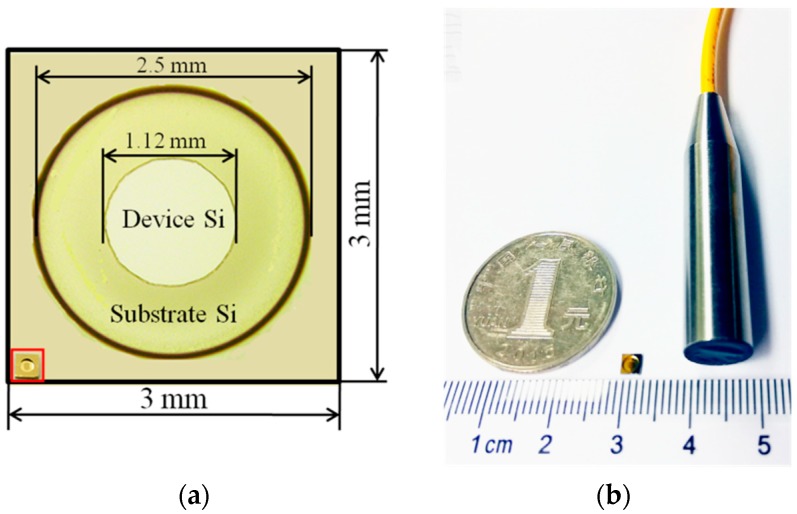
Photographs of (**a**) SOI diaphragm structure and the sensor chip; (**b**) a packaged extrinsic Fabry–Perot interferometric (EFPI) sensor probe.

The microscope image of a fabricated sensor chip is shown in Figure 4a. Finally, the sensor probe is assembled with good alignment accuracy between the sensor chip and the fiber’s end face, which is ensured by the stepped hole and a near-infrared spectrometer. Figure 4b shows a photograph of the packaged sensor probe. A 10-mm-diameter stainless steel tube is used to ensure its rigidity; however, it is easy to tailor the sensor package with a smaller size, because the maximum diameter of the sensor chip is only $3\sqrt{2}$ mm. Considering the environmental influences, the ultrasonic sensor is protected from contamination and moisture by a sealing film to achieve a more reliable measurement. The temperature variation in the electrical equipment may lead to the thermal expansion deformation of the diaphragm structure. The thermal simulation result by ANSYS shows that when the environmental temperature rises from 22 to 85 °C, the expansion along the *z* direction of the 5-μm-thick diaphragm is only 1 nm. This small cavity length change will not influence the linear performance of the sensor. In addition, the residual stress in the diaphragm may lead to a curved diaphragm surface and correspondingly a non-uniform FP cavity. However, the central reflecting area of diaphragm is only 9 μm according to the fiber core diameter. Thus, a central roughness less than 140 nm can still ensure that 95% of the reflected light re-enters into the fiber to maintain a high optical throughput [13].

As shown in Table 1, this work is compared with the four latest studies of EFPI ultrasonic sensors. It is obvious that the cavity length is mostly adjusted by a translation stage, which is time-consuming and it is difficult to maintain a desired length when the sensor probe is assembled. Therefore, a novel stepped hole is designed in our sensor chip for not only fixing the fiber end easily but also the consistency of the cavity length of all of the chips over the whole 4-inch wafer. The 100-nm-thick graphene diaphragm in [12] has a high sensitivity of 1100 nm/Pa but may limit the ultrasound response in PDs detection because of its small resonant frequency of 10 kHz. In addition, by utilizing the microelectromechanical system (MEMS) manufacturing process on a silicon-on-insulator (SOI) wafer, it is more productive and of a lower cost than the polymer or graphene diaphragm fabrication process. More than 500 micromechanical silicon sensing chips can be shaped on one 4-inch wafer.

## 4. Experimental Investigation and Results

**Figure 5:**
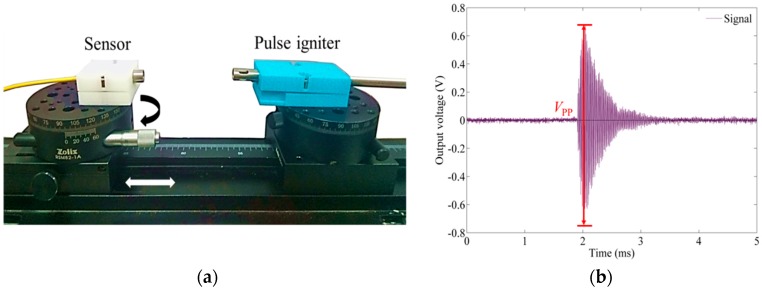
(**a**) Photograph of the experimental setup; (**b**) The detected ultrasonic signal from the pulse igniter.

When a PD occurs on the ageing insulation system of assets, the generated ultrasonic wave transmits through an air path from the PD site to the outside of the instrument and then can be detected externally [24,25]. Therefore, in this experiment, the discharge from a pulse igniter was set to occur in the air. To perform the measurements based on the various incident angles and distances, as shown in Figure 5a, the sensor probe is fixed on a precise rotary stage and is stacked on an optical slide rail. Both the sensor probe and pulse igniter are aligned at the same height. The distributed feedback (DFB) fiber laser is emitted by a direct-current-regulated power supply. A high-speed amplifier (TPIN-LW-M, COSC, Beijing, China) with a photodiode is used to convert the light signals to voltage with noise of ±5 mV. The responsivity of the InGaAs photodiode in this device is 0.9 A/W and the conversion gain of the amplifier is 0.6 × 10^6^ V/A. Considering that most PDs occur in the frequency range of 40–300 kHz [19], the sampling rate of the DAQ device (NI 6351) is set to 1 MHz. The processing circuit was packaged in the electrical enclosure to ensure a low-level interference. Figure 5b shows an exemplified PD ultrasonic signal detected by the EFPI sensor and low noise around 20 mV from the whole sensing system. Because each discharge from the pulse igniter is random and not totally the same in energy, such as the PDs happening in the electrical equipment, at least 10 effective discharges are measured at each source location. Then, the peak-to-peak voltage (*V*_pp_) values of these discharges are calculated and averaged to ensure the accuracy for the measurement of the directional response.

**Figure 6:**
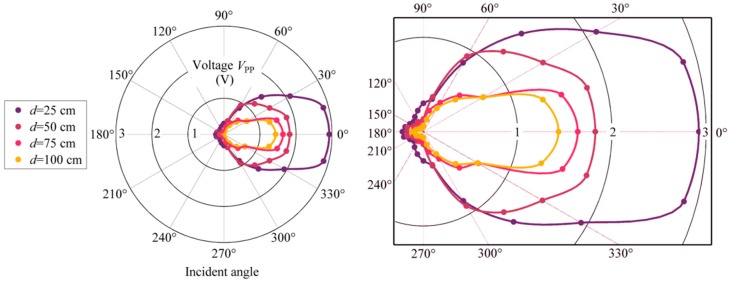
Ultrasonic signals detected at different incident angles and distances.

Figure 6 shows the *V*_pp_ values of the sensing system for different ultrasound incident angles α varying from 0 to 360° with a step of 15°. The angle-dependent measurements are taken in four different distances *d* between the discharge source and the sensor. The measured voltages at 25, 50, 75, and 100 cm demonstrate a similar variation trend, and each fitting curve shows an approximately symmetric distribution along the horizontal axis. The received signal within the ±45° incident range at 50 cm has a relatively flat response varying by 3.58 dB and 5.90 dB within ±60°, which is close to the results of an epoxy-encapsulated EFPI sensor [19]. However, utilizing rubber or polymer as the encapsulation material can create a wider angular response because of their smaller acoustic impedance than the air–steel interface. There are repeated results showing that the weakest signal is mostly detected at 135° but not the backward direction of 180°. However, there is no reflective medium in front of the sensor to make an echo. One possible explanation is that the polymer adhesive used at the end of the stainless steel tube to seal the gap has better permeability to ultrasound than the stainless steel. Therefore, a part of the backward incident ultrasound waves transmits through the adhesive into the sensor chip and makes the diaphragm vibrate, while the ultrasound wave for other incident angles, such as 135°, meets the tube surface firstly and leads to a huge attenuation due to the mismatch of acoustic impedance. In addition, the slope on the tube end may also plays a role in decreasing acoustic pressure. Nonetheless, the sensor is able to recognize weak signals of discharges from all incident angles within 1 m, especially in the range of ±60°. The results also exhibit an optimized sensor array: only three sensors, which are embedded in the electrical equipment, are required to realize on-site monitoring and accurate localization.

**Figure 7:**
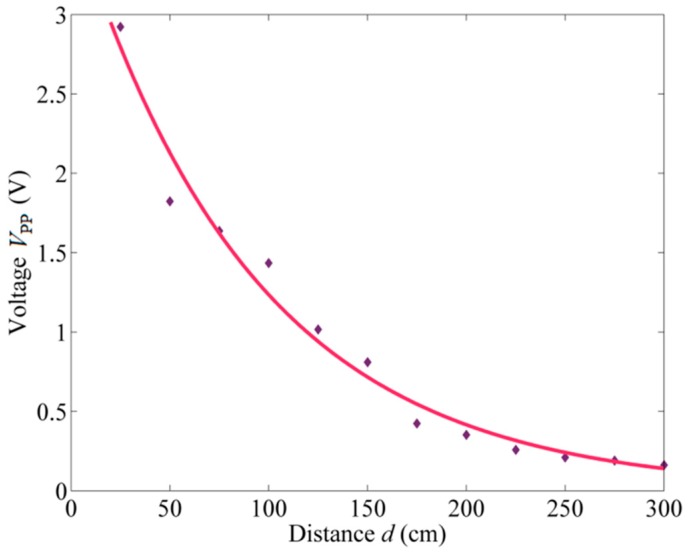
Ultrasonic signals detected at different linear distances.

Figure 7 shows the *V*_pp_ values of the detected signals based on different distances *d* varying from 25 cm to 300 cm with a step of 25 cm. The measured data indicate an exponential decay as the distance increases. Then, a fitting curve is constructed utilizing the MATLAB fitting toolbox, and the result is:

(8) $$V_{\mathrm{pp}}=3.67\cdot e^{-0.01089d},\;{(R}^{2}=0.9772)$$

which accords with the attenuation property of ultrasound waves in the air [26].

**Figure 8:**
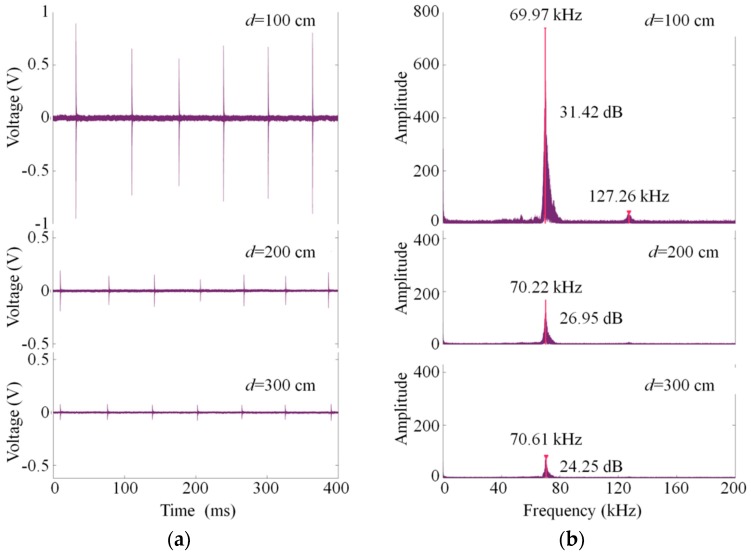
Ultrasonic signal waveforms at *d* = 100 cm, 200 cm, and 300 cm in (**a**) the time domain; (**b**) the frequency domain.

The ultrasonic signals of discharges at 100, 200, and 300 cm are shown in Figure 8a, respectively. As the discharge source is 300 cm to the sensor, there is a distinct signal that is as great as 8 times the background noise, which indicates the high sensitivity of the ultrasonic sensor. The amplitude-frequency responses are computed by the Fast Fourier Transform (FFT) function in MATLAB and plotted in Figure 8b. All of the detected ultrasound signals show a spike in magnitude around the signal’s frequency components of 70 kHz. The other smaller peak amplitude can be observed around 127 kHz when the discharge source is closer. Moreover, the signal-to-noise ratio (SNR) has been marked on each figure. The SNR of 31.42 dB at *d* = 100 cm is slightly higher than that of the reported fiber-optic ultrasonic sensor [27] and shows a possibility for the detection of weak sound signals at a further distance.

The directional-dependence sensitivity is an intrinsic form of performance determined by the vibrating structure and the package design of the sensor. Thus, the transformer environment of PDs activity is simplified in this work. For a practical application, benefitting from its small size, intrinsic safety, and immunity from electromagnetic interference, this fiber-optic ultrasonic sensor can be installed on the transformer tank wall to achieve better performance than external detection. Moreover, the dual-sensor system, composed of an ultrasonic sensor and an ultrahigh frequency sensor, is expected to improve measurement accuracy and reliability.

## 5. Conclusions

In conclusion, the directional sensitivity of the fiber-optic EFPI ultrasonic sensor according to the incident angle and linear distance is investigated. The sensor, which has a high sensitivity of 733 nm/kPa, exhibits a response range of ±60° with a 5.90 dB amplitude fluctuation at 50 cm. A SNR of 31.42 dB at 1 m and the noise below 20 mV of the sensing system show its capability to detect a weak discharge signal from 3 m away in the air. The results also provide a perspective on the optimization of the sensor package and the further configuration of the sensor array for PD detection.

## Figures and Tables

**Table 1:** Comparison with the four latest EFPI ultrasonic sensors.

Sensor Fabrication		Ref. [10]	Ref. [12]	Ref. [21]	Ref. [9]	Present Work
EFPI diaphragm	material	PPS *	graphene	silica	PTFE *	silicon
	thickness	1.2 μm	0.1 μm	75 μm	30 µm	5 μm
	diameter	4.9 mm	0.125 mm	1.8 mm	2 mm	1.12 mm
	resonant frequency	40 kHz	10 kHz	252 kHz	300 kHz	60 kHz
	sensitivity	/	1100 nm/kPa	3.9 nm/kPa	/	733 nm/kPa
fabrication process	FP cavity length control	nanometer displacement table	translation stage	translation stage	micrometer	self-adjusted by the stepped hole
	assembling	ferrule, capillary, and fiber fixed by epoxy	ferrule and fiber held by curable gel, grapheme diaphragm transferred from the sample	silica diaphragm, ferrule, and sleeve with fiber bonded by thermal laser welding	PTFE diaphragm, tubes, and fiber bonded by glue	sensor chip, tube, and fiber fixed by glue
	productivity	low	low	low	low	high

* Where PPS is polyphenylene sulfide and PTFE is polytetrafluoretyhylene.
